# Social cognition in the blind brain: A coordinate‐based meta‐analysis

**DOI:** 10.1002/hbm.25289

**Published:** 2020-12-15

**Authors:** Maria Arioli, Emiliano Ricciardi, Zaira Cattaneo

**Affiliations:** ^1^ Department of Psychology University of Milano‐Bicocca Milan Italy; ^2^ IMT School for Advanced Studies Lucca Lucca Italy; ^3^ IRCCS Mondino Foundation Pavia Italy

**Keywords:** action observation network, activation likelihood estimation, blind, functional magnetic resonance imaging, meta‐analysis, social cognition

## Abstract

Social cognition skills are typically acquired on the basis of visual information (e.g., the observation of gaze, facial expressions, gestures). In light of this, a critical issue is whether and how the lack of visual experience affects neurocognitive mechanisms underlying social skills. This issue has been largely neglected in the literature on blindness, despite difficulties in social interactions may be particular salient in the life of blind individuals (especially children). Here we provide a meta‐analysis of neuroimaging studies reporting brain activations associated to the representation of self and others' in early blind individuals and in sighted controls. Our results indicate that early blindness does not critically impact on the development of the “social brain,” with social tasks performed on the basis of auditory or tactile information driving consistent activations in nodes of the action observation network, typically active during actual observation of others in sighted individuals. Interestingly though, activations along this network appeared more left‐lateralized in the blind than in sighted participants. These results may have important implications for the development of specific training programs to improve social skills in blind children and young adults.

## Graphical abstract

Social cognition skills are typically acquired on the basis of visual information. Here we provide a meta‐analysis of neuroimaging studies reporting brain activations associated to the representation of self and others' in early blind individuals and in sighted controls. Our results indicate that early blindness does not critically impact on the development of the “social brain,” with social tasks performed on the basis of auditory or tactile information driving consistent activations in nodes of the action observation network, typically active during actual observation of others in sighted individuals.

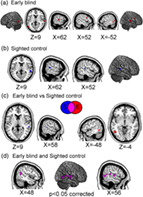

## 1 INTRODUCTION

Social cognition refers to a complex set of neurocognitive processes underlying the individuals' ability to decode others' mind to plan actions in the social environment (Arioli, Crespi, & Canessa, 2018; Todorov, Harris, & Fiske, 2006). The ability to perceive social information (e.g., face expression, posture, voice, action goal) and draw inferences on others' mental states is crucial for survival, cooperation in social communities, communication and culture (Dolan, 2002). A fundamental prerequisite of social cognition is the ability to differentiate between objects (whose movement is completely explained by physical forces) and human beings (whose behavior is characterized by motivations, emotions and believes, which make their actions not completely predictable) (Fiske & Taylor, 2013; Vogeley, 2017). Social stimuli, thus, seem to represent a qualitatively different perceptual category, mediated by dedicated neurocognitive mechanisms (Maurer, Grand, & Mondloch, 2002). The prototypical example in this view is the processing of human faces (Said, Haxby, & Todorov, 2011) and human bodies in dedicated neural circuitries (i.e., the occipital face area and the extrastriate body area in the occipitotemporal cortex, and the fusiform face area and the fusiform body area in the fusiform gyrus; see Bernstein, Erez, Blank, & Yovel, 2018; Peelen & Downing, 2007).

The category of social stimuli potentially includes any kind of information concerning social entities, behavior and words referring to them (Arioli, Gianelli, & Canessa, 2020). The visual processing of social stimuli, and human faces in particular, seems to represent the richest source of information in everyday social life: eyes represent the most informative social stimuli (Adams & Nelson, 2016), gaze perception is considered a crucial step for mentalizing (Baron‐Cohen, Jolliffe, Mortimore, & Robertson, 1997) and emotional expressions, produced by the contraction of facial muscles, provide essential social information (Todorov, Olivola, Dotsch, & Mende‐Siedlecki, 2015). Body postures and gestures are also critical in conveying emotional states (for a review, see de Gelder, de Borst, & Watson, 2015) and the study of body emotional perception, both in healthy individuals and patients, has gained increasing attention in recent years (e.g., Ferrari, Ciricugno, Urgesi, & Cattaneo, 2019; Ferrari, Papagno, Todorov, & Cattaneo, 2019; Lenzoni et al., 2020; Soria Bauser, Thoma, & Suchan, 2012; Thoma et al., 2014; Thoma, Soria Bauser, & Suchan, 2013). Part of the social information conveyed by visual cues—such as one's emotional state—may also be acquired via non‐visual sensory modalities: for instance, voice intonation and loudness, which are features of speech, collectively termed as “prosody”, are important cues in understanding the speaker's emotional state (see Mitchell, Elliott, Barry, Cruttenden, & Woodruff, 2003). Nonetheless, other cues important for social interactions (such as social gestures) can only be perceived visually. Not surprising, most of the available literature in social neuroscience has focused on the neural processing of *visual* social stimuli. Two brain systems, the action observation/mirror system and the theory of mind/mentalizing system, are responsible for the perception and representation of other individuals' states, actions and intentions (Van Overwalle & Baetens, 2009). Neuroimaging studies identify fronto‐parietal and occipito‐temporal regions collectively termed the action observation network (AON) as critically involved in processing others' actions and the meaning underlying them, via automatic simulation routines (Caspers, Zilles, Laird, & Eickhoff, 2010). This system allows us to internally, rapidly and intuitively simulate observed actions within our own sensorimotor system, providing an enriched “understanding” of another person's goals and intentions, on the basis of low‐level behavioral input (Caspers et al., 2010; Iacoboni et al., 2005). In particular, observing actions recruits the superior and middle temporal gyrus (STG and MTG), inferior parietal lobule (IPL) and inferior frontal gyrus (IFG) (Gardner, Goulden, & Cross, 2015). In contrast, the mentalizing system allows to make inferences on others' mental and affective states (Molenberghs, Johnson, Henry, & Mattingley, 2016), involving the medial precuneus and temporo parietal junction (TPJ), as well as the ventro‐medial and dorso‐medial prefrontal cortex (Amodio & Frith, 2006). Consistent evidence entails gaze perception as one of the key factors for mentalizing ability (Calder, Young, Keane, & Dean, 2000). Although the AON and the mentalizing networks are mainly distinct, they are likely to play a complementary role during social interactions, and recent meta‐analytic evidence suggests a common involvement of certain brain regions in both, such as the right pSTS—bordering the TPJ (Arioli & Canessa, 2019). Particularly, both systems are engaged during social interactions: the mirror system is responsible for the preparation of our own actions and the simulation of others' actions, while the mentalizing system allows to represent others' intentions, by drawing the capacity to understand the others' thoughts and beliefs (Sperduti, Guionnet, Fossati, & Nadel, 2014).

Considering the great role of vision in supporting human social cognition, a critical issue is the impact that blindness, and in particular the lack of vision since birth, may have on the development of social cognition, both at the functional and neural level. Blindness may indeed affect the development of emotional responsiveness and social skills, possibly predisposing to features of social isolation (including autism, e.g., Brunes, Hansen, & Heir, 2019; Hobson, Lee, & Brown, 1999), as well‐known by blind educators that are challenged with the need to develop effective programs to promote social skills in blind children and youngsters (see Sacks, Kekelis, & Gaylord‐Ross, 1992; Sacks & Wolffe, 2006). Accordingly, studies carried out in laboratory contexts suggest that blind children show impairment in several social abilities, such as representing others' mental and affective states (Brambring & Asbrock, 2010; Dyck, Farrugia, Shochet, & Holmes‐Brown, 2004; Green, Pring, & Swettenham, 2004) and acting social interactions (Pérez‐Pereira & Conti‐Ramsden, 2013; Tadic, Pring, & Dale, 2010). Blind children seem also to exhibit a more limited repertoire of facial expressions compared to sighted children (Tröster & Brambring, 1992; see also Webb, 1977). The acquisition of verbal skills may reduce the impact of early visual deprivation on social capacities (Bedny & Saxe, 2012), with blind adults being able to understand other individuals' emotions and mental states in a way comparable to that of sighted individuals (e.g., Gamond, Vecchi, Ferrari, Merabet, & Cattaneo, 2017; Oleszkiewicz, Pisanski, & Sorokowska, 2017). Nonetheless, it is likely that social cognition may be mediated by at least partially different strategies and mechanisms in blind and sighted individuals. For example, blind individuals rely on partially different strategies in making impressions about social actors (Ferrari, Vecchi, Merabet, & Cattaneo, 2017), have difficulties in the posing of emotional expressions (Valente, Theurel, & Gentaz, 2018) and do not seem to show valence‐dependent hemispheric lateralization when processing emotions (Gamond et al., 2017). Whereas a consistent body of neuroimaging research has investigated how blindness affects at the neural level perceptual and cognitive processes, only a few studies have systematically investigated how blindness affects the way the brain mediates social processes. Two pioneering fMRI studies first showed that nonvisual modalities may still drive the development of the cortical networks underlying action recognition and Theory of Mind processes (e.g., Bedny, Pascual‐Leone, & Saxe, 2009; Ricciardi et al., 2009). While these observations suggest that the large‐scale functional organization of the “social brain” is maintained in congenitally blind individuals, there is also evidence that brain networks specifically devoted to face or voice processing may develop differently in the absence of visual experience (e.g., Holig, Focker, Best, Roder, & Buchel, 2014; Pietrini et al., 2004; Van Ackeren, Barbero, Mattioni, Bottini, & Collignon, 2018).

Meta‐analyses are a useful approach that allows to highlight the consistency of neural pattern across different experimental studies, by integrating data into a unique statistical analysis. Whereas a recent meta‐analysis by Zhang et al. (2019) has clarified the neurocognitive mechanisms underlying language, spatial and object processing in early blind individuals, a comparable approach in the domain of social cognition has never been implemented. In light of the above, in this study we implemented a quantitative meta‐analysis of the available neuroimaging literature to provide more solid evidence on: (a) the brain regions associated with the neural representation of others in early blind individuals and (b) the specific brain activation in early blind individuals compared with sighted control individuals. Specifically, we employed activation likelihood estimation (ALE) in order to draw convergence across neuroimaging experiments on others' representation in early blind individuals.

## 2 MATERIALS AND METHODS

### 2.1 Rationale of the meta‐analytic approach

We took a quantitative meta‐analytic approach to investigate the neural representation of others in early blind individuals and to unveil which brain regions are selectively recruited in early blind compared with sighted control individuals. Critically, with this approach we can overcome the typical limitations inherent in single neuroimaging studies, for example, sensitivity to experimental and analytic procedures, lack of replication studies, as well as small sample size (Carp, 2012). These constraints are known to increase the likelihood of false negatives (Button et al., 2013), thus pushing researchers toward procedures which, conversely, might promote false positives (Eklund, Nichols, & Knutsson, 2016; Muller et al., 2018). We thus aimed to identify the brain regions *consistently* associated with the neural coding of others in early blind individuals, over and beyond this process in sighted control groups. This goal was pursued with ALE, a coordinate‐based meta‐analytic approach using the MNI coordinates of peak locations to summarize and integrate published findings (Turkeltaub, Eden, Jones, & Zeffiro, 2002). Thus, we ran two separate ALE analyses: one for blind individuals and one for sighted control individuals. After that, contrast analyses were conducted between the blind and the sighted control groups. In particular, we aimed to investigate brain activations related to others' representation irrespective of the input sensory modality (i.e., tactile or auditory), the stimulus type (i.e., vocalizations, sound of actions, words, 3D models of faces to be tactically explored, etc.), and the specific employed task.

All the inclusion criteria for each dataset were selected by the first author, and then checked by the other authors. This procedure, entailing a double check by independent investigators, was aimed to reduce the chances of a selection bias (Muller et al., 2018).

### 2.2 Literature search and study selection: The representation of others in blind and sighted control individuals

**FIGURE 1:**
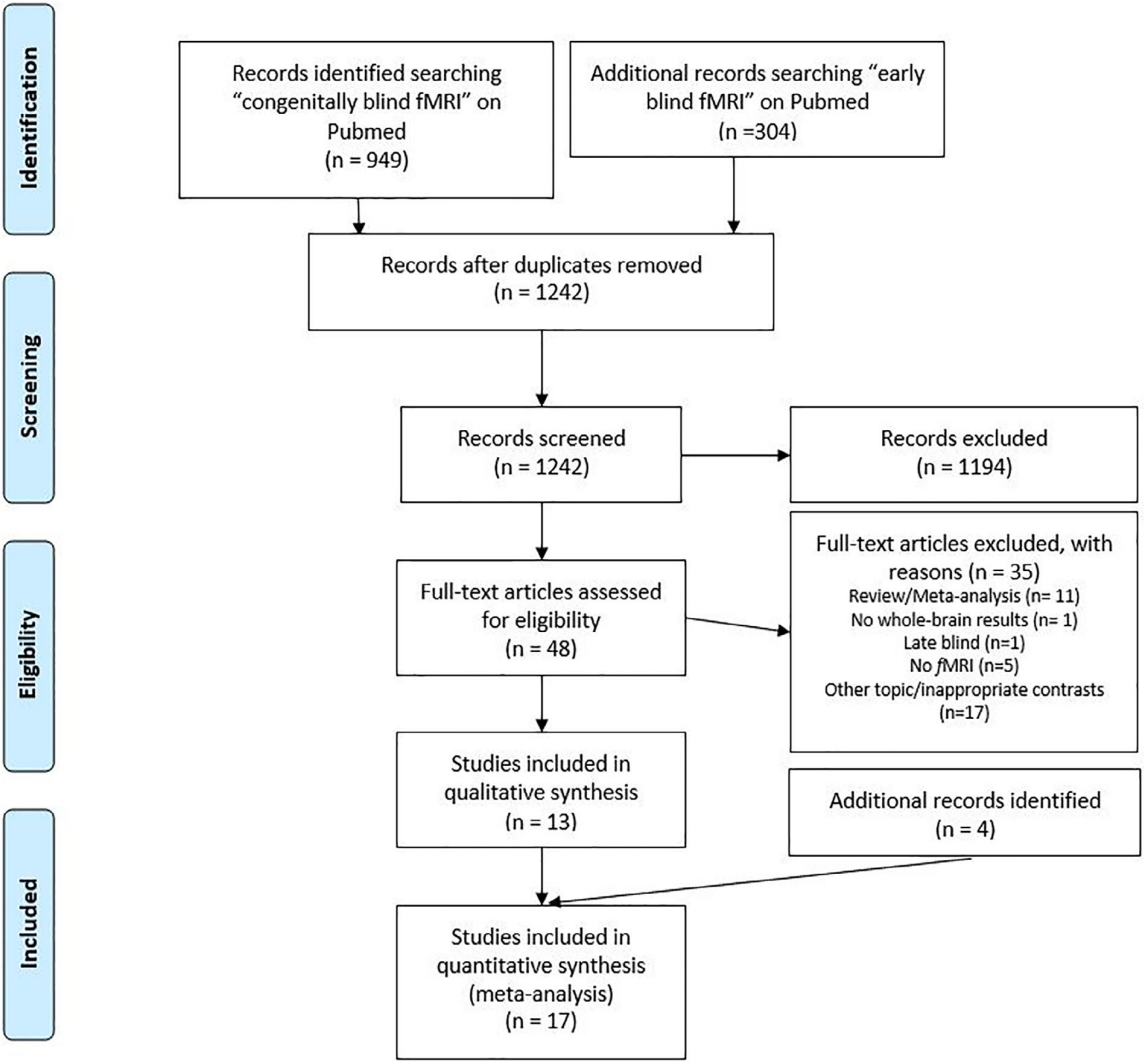
Flowchart of literature search and selection process

We started our survey of the relevant literature by searching for “early blind *f*MRI” and “congenitally blind *f*MRI” on Pubmed (https://www.ncbi.nlm.nih.gov/pubmed/). The preliminary pool of 1,242 studies, after duplicates removed, was first screened by title, and then abstract. We retained only those studies fulfilling the following selection criteria (see Figure 1 for the detailed study selection process):

1. studies written in English language;
2. empirical *f*MRI studies, while excluding review and meta‐analysis articles and those employing other techniques, to ensure comparable spatial and temporal resolution;
3. studies reporting whole‐brain activation coordinates, rather than results limited to regions of interest (ROIs) or using small volume corrected (SVC) analyses. Studies based on ROI or SVC analyses should be excluded from meta‐analyses (Muller et al., 2018), because a prerequisite is that convergence across experiments is tested against a null‐hypothesis of random spatial associations across the entire brain under the assumption that each voxel has the same a priori chance of being activated (Eickhoff, Bzdok, Laird, Kurth, & Fox, 2012);
4. studies focused on early blind participants, rather than on late blind participants. In fact, we decided to focus only on early visual deprivation since neural plasticity phenomena critically depend on age at blindness onset, with consistent evidence showing that following early visual experience, several brain areas maintain a vision‐dominated response pattern as an outcome of the early visual experience (e.g., Bedny, Konkle, Pelphrey, Saxe, & Pascual‐Leone, 2010; Voss, Gougoux, Zatorre, Lassonde, & Lepore, 2008; see also Cattaneo, Vecchi, Monegato, Pece, & Cornoldi, 2007);
5. studies investigating brain activity related to representation of other individuals (including mental states, physical traits, and human action recognition, see below for examples) as opposed to conditions where no representation of other individuals was involved (such as studies assessing memory capacities, language, or spatial processing). To this purpose, we selected contrasts requiring participants to attend to stimuli aimed to elicit a representation of other individuals and contrasting this kind of representation with baseline conditions where there was no human representation. The included studies ranged from those requiring participants to represent others' mental states (Bedny et al., 2009) to studies comparing responses to human voice processing versus object sounds (Dormal et al., 2018), to studies comparing haptic recognition of basic facial expressions versus object discrimination (Kitada et al., 2013), as well as studies assessing neural activations in response to sounds produced by human motion (such as footsteps, Bedny et al., 2010) or hand‐actions (such as cutting paper with scissors, Ricciardi et al., 2009) compared to non‐human environmental sounds.

The articles that were excluded, based on titles and abstracts, included review or meta‐analysis studies (31 studies), single case studies (100 studies), studies not including a blind group (12 studies), studies assessing other (non‐social) cognitive processes (such as spatial representation) in early blind subjects (137 studies) or in subjects with cortical visual impairment (34 studies) or in sighted individuals (646 studies), studies written in non‐English language (49 studies) and studies not employing task‐based *f*MRI technique (185 studies; of which 11 studies investigated non‐social processes in blind subjects, 173 focused on non‐social processes in sighted subjects and 1 study studied social processes in sighted individuals).

From the remaining 48 articles we retained only those fulfilling the above selection criteria (see Figure 1) and, thus, we excluded review and meta‐analysis articles (11 articles) and studies employing non‐fMRI technique (5 article); studies using ROIs or SVC analyses (1 study); studies focused on late blind participants (1 study) and studies that did not focus on others' representation (17 studies).

We included studies fulfilling the above criteria regardless of: (a) tested sensory modality (e.g., auditory or haptic), (b) experimental paradigm (e.g., memory or identification tasks). Our aim was indeed to pool across different experimental paradigms to ensure both generalizability and consistency of results, within the “others' representation > non‐human representation” comparison inherent in our research question (Radua & Mataix‐Cols, 2012). This selection phase resulted in 13 studies (out of 374) fulfilling our criteria. In some cases, we directly contacted the authors to have clarification and more information regarding their study.

We then expanded our search for other potentially relevant studies by carefully examining both the studies quoting, and those quoted by, each of these papers, alongside a recent meta‐analysis on the cognitive processes of blind individuals (Zhang et al., 2019). This second phase highlighted four further studies fitting our search criteria.

This procedure led to include in the main ALE meta‐analysis on blind participants 17 previously published studies (see Table 1), resulting from 17 experiments (individual comparisons reported) with 212 subjects and 276 foci. The same procedure led to include 16 previously published studies, resulting from 16 experiments, with 267 subjects and 331 foci in the ALE analysis regarding the sighted control group (see Table 1). This number of contrasts is in line with the recent prescriptions for ALE meta‐analyses (Eickhoff et al., 2016; Muller et al., 2018), and ensures that results would not be driven by single experiments (see also Zhang et al., 2019).

**TABLE 1:** Overview of the 17 studies included in the meta‐analysis on the neural representation of other individuals in both blind and sighted groups

N	First author/ year	Sub	Stimuli	Task	Contrast	Foci EB	Foci SC
1	Bedny et al. (2009)	10 EB + 22 SC	Auditory (stories recorded by female speaker)	True/false question task	Mental stories (voice) > noise control condition; mental stories > physical stories	11	17
2	Bedny et al. (2010)	10 EB	Auditory (sounds)	Motion detection task	Human sound (footsteps) > non‐human sound (tones)	5	—
3	Bedny and Saxe (2012)	10 EB + 20 SC	Auditory (words)	Semantic judgment task	Actions verbs > natural inanimate objects; mental verbs > animal nouns	1	8
4	Bedny et al. (2015)	19 EB + 20 SC	Auditory (stories recorded by female speaker)	“Does this come next?” task	Spoken language > music	6	4
5	Dormal et al. (2018)	16 EB + 15 SC	Auditory (voices)	Repetition detection task	Voice > scrambled voice; voice > baseline (silence)	23	27
6	Fairhall et al. (2017)	7 EB + 15 SC	Auditory (vocalizations and statements)	One‐back repetition detection task	Verbal emotional stimuli (voice) > baseline; vocalization stimuli > baseline	57	63
7	Gougoux et al. (2009)	5 EB + 14 SC	Auditory (voices)	Implicit task (listening)	Vocal stimuli > non‐vocal stimuli	3	4
8	Holig et al. (2014)	12 EB + 11 SC	Auditory (voices)	Voice identity recognition	Voice > rest period	90	53
9	Kitada et al. (2013)	17 EB + 22 SC	Haptic (plastic casts)	Haptic identification task	Face expressions > shoes	3	13
10	Kitada et al. (2014)	24 EB + 28 SC	Haptic (plastic casts)	Haptic identification task	Hands > nonbiological control objects	18	23
11	Lewis et al. (2011)	10 EB + 14 SC	Auditory (sounds)	Auditory identification task	Human action sound > unrecognizable real‐world sounds	5	4
12	Ma and Han (2011)	19 EB + 19 SC	Auditory (statements)	Judgment task	Others' judgment task > valence judgment task	2	2
13	Noppeney et al. (2003)	11 EB + 12 SC	Auditory (words)	Voice identity recognition	Hand action words > other semantic types	1	1
14	Ricciardi et al. (2009)	8 EB + 14 SC	Auditory (sounds)	Sound recognition	Sounds of hand‐executed familiar actions > environmental sounds	3	6
15	Striem‐Amit and Amedi (2014)	7 EB + 7 SC	Auditory (sounds)	Visual‐to‐auditory sensory‐substitution device (SSD, the “vOICe”)	Body shape > baseline	12	6
16	van den Hurk et al. (2017)	14 EB + 18 SC	Auditory (words)	One‐back task	Face and body auditory conditions > baseline	15	72
17	Hwang and Matsumoto (2016)	13 EB + 16 SC	Auditory (words)	Size judgment task	Face parts and celebrity > places (both famous and daily)	21	28
	Total sub:	212 EB + 267 SC			Total foci:	276	331

*Note*: The majority of these studies employed auditory stimuli (15/17, hence almost 90% of the studies we included), while only two studies used haptic stimuli (Kitada et al., 2013, 2014).

Abbreviations: EB, early blind individuals; N, progressive study number; SC, sighted control individuals; Sub, subjects.

Importantly, the inclusion of multiple contrasts/experiments from the same set of subjects can generate dependence across experiment maps and thus decrease the validity of meta‐analytic results. To prevent this issue, we adjusted for within‐group effects by pooling the coordinates from all the relevant contrasts of a study into one experiment (Turkeltaub et al., 2002).

### 2.3 Activation likelihood estimation

We performed two distinct ALE analyses, using the GingerALE software (Eickhoff et al., 2009), to identify consistently activated regions associated with the representation of others in both blind and sighted control groups. We followed the analytic approach previously described by Arioli and Canessa (2019), based on Eickhoff et al. (2012). In both meta‐analyses, activation foci were initially interpreted as the centres of three‐dimensional Gaussian probability distributions, to capture the spatial uncertainty associated with each individual coordinate. All coordinates were reported or converted into MNI space, using the automatic routine implemented in GingerALE. The three‐dimensional probabilities of all activation foci in a given experiment were then combined for each voxel, resulting in a modeled activation (MA) map. The union of these maps produces ALE scores describing the convergence of results at each brain voxel (Turkeltaub et al., 2002). To distinguish “true” convergence across studies from random convergence (i.e., noise), the ALE scores are compared with an empirically defined null distribution (Eickhoff et al., 2012). The latter reflects a random spatial association between experiments, with the within‐experiment distribution of foci being treated as a fixed property. A random‐effects inference is thus invoked, by focusing on the above‐chance convergence between different experiments, and not on the clustering of foci within a specific experiment. From a computational standpoint, deriving this null hypothesis involved sampling a voxel at random from each MA map, and taking the union of the resulting values. The ALE score obtained under this assumption of spatial independence was recorded, and the permutation procedure iterated 100 times to obtain a sufficient sample of the ALE null distribution. The “true” ALE scores were tested against the ALE scores obtained under the null distribution and thresholded at *p* < .001, corrected for cluster‐level family wise error, and the cluster level threshold was set at *p* < .05, to identify above‐chance convergence in each analysis (Eickhoff et al., 2012).

The resulting maps were then fed into direct comparisons and conjunction analyses, within GingerALE, to unveil the common and specific brain activations between the early blind and sighted control individuals. A conjunction image was created, using the voxel‐wise minimum value of the included ALE images, to display the similarity between datasets (Eickhoff et al., 2011). In the same analysis, two ALE contrast images were created and compared by directly subtracting one input image from the other. To correct for sampling errors, GingerALE creates such data by pooling the foci in each dataset and randomly dividing them into two new groupings equivalent in size to the original datasets. An ALE image is created for each new dataset, then subtracted from the other and compared with the true data. Permutation calculations are then used to compute a voxel‐wise *p* value image indicating where the values of the “true data” fall within the distribution of values in any single voxel. To simplify the interpretation of ALE contrast images, significant ALE subtraction scores were converted to *Z* scores. For between‐group contrast analyses, we used a threshold set at *p* < .05, corrected for false discovery rate, and minimum volume size of 100 mm^3^.

## 3 RESULTS

### 3.1 Others' representation in early blind individuals

**FIGURE 2:**
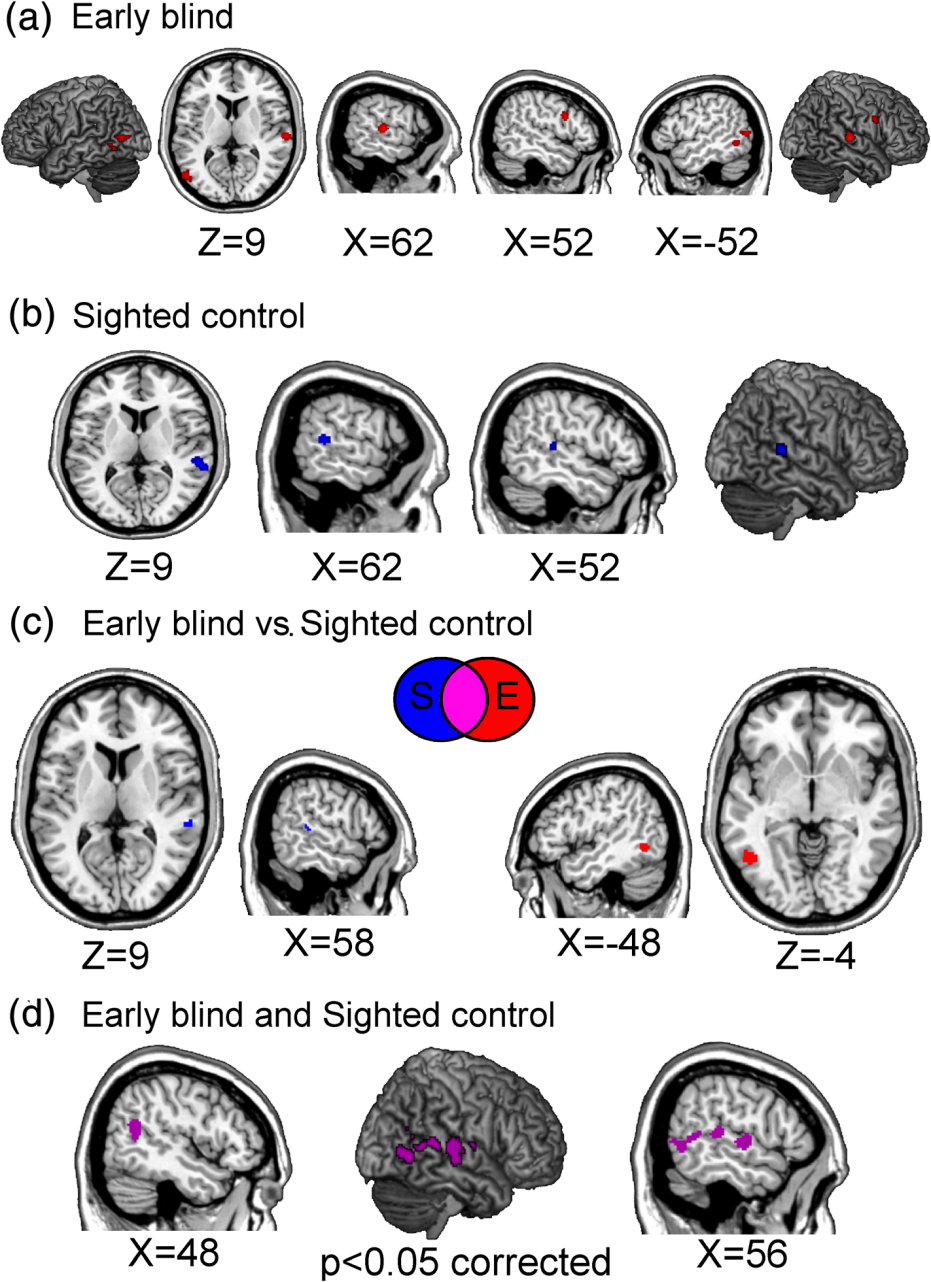
Neural processing of others in early blind (EB) individuals, sighted control (SC) individuals and differences between EB and SC participants. The figure reports the brain structures consistently associated with processing other individuals in EB (a), and SC subjects (b), and the results of direct comparisons and conjunction analysis between the meta‐analyses separately performed on the two different groups (c); this analysis reported no significant common activation to the processing of others in EB and SC, likely due to the low number of studies included in each meta‐analysis (17 studies for EB and 16 studies for the SC group). The last panel (d) shows the results of a third meta‐analysis carried out considering all studies (both on EB and SC, 33 experiments included) to unveil brain regions consistently engaged during social processing in both groups. All the reported activations survived a statistical threshold of *p* < .05 corrected for multiple comparisons

Activations associated with the neural processing of others in early blind individuals encompassed the regions typically associated with the AON. These included the posterior portion of the right inferior frontal gyrus, as well as the inferior and middle temporal cortex, extending in the right superior temporal sulcus (STS) and the left fusiform gyrus (see Table 2 and Figure 2a).

**TABLE 2:** Neural bases of others' representation in early blind subjects

Cluster #	Cluster size (mm^3^)	Brain region	*x*	*y*	*z*
1	872	Right superior temporal gyrus	62	−22	6
2	720	Left fusiform gyrus	−52	−58	−4
		Left middle temporal gyrus	−60	−56	0
3	720	Right inferior frontal gyrus	52	8	28
4	712	Left middle temporal gyrus	−54	−70	8

*Note*: From left to right, the table reports the size (in mm^3^), stereotaxic coordinates of local maxima and anatomical labeling of the clusters which were consistently associated with representing others in early blind subjects.

The lack of consistent activation in the parietal cortex, a key node of the AON, is possibly due to the low number of studies specifically focusing on hand representation in our database (see Section 4 and Table 1).

### 3.2 Others' representation in sighted control individuals

Activations associated with representing others in sighted control groups involved the right superior and middle temporal gyri (see Table 3 and Figure 2b).

**TABLE 3:** Neural bases of others' representation in the sighted control group

Cluster #	Cluster size (mm^3^)	Brain region	*x*	*y*	*z*
1	1,032	Right middle temporal gyrus	62	−38	8
		Right superior temporal gyrus	56	−32	8

*Note*: From left to right, the table reports the size (in mm^3^), stereotaxic coordinates of local maxima and anatomical labeling of the clusters which were consistently associated with representing others' in sighted subjects.

### 3.3 Others' representation in early blind and sighted control individuals

A conjunction analysis highlighted no significant common activation to the processing of other individuals in early blind and sighted control subjects (Table 4).

**TABLE 4:** Brain regions showing common and specific activations in the early blind and sighted groups during representation of other individuals

Early blind and sighted
N.A.

Early blind > sighted					
Cluster #	Cluster size (mm^3^)	Brain region	*x*	*y*	*z*
1	616	Left fusiform gyrus	−48	−58	−8
		Left middle temporal gyrus	−47	−56	−4

Sighted > early blind					
Cluster #	Cluster size (mm^3^)	Brain region	*x*	*y*	*z*
1	104	Right middle/superior temporal gyrus	58	−34	6

*Note*: From left to right, the table reports the size (in mm^3^), stereotaxic coordinates of local maxima and anatomical labeling of the clusters which were specifically activated in early blind and sighted groups.

The lack of common neural activations between the two groups during social processing was somehow unexpected and is probably guided by the low number of studies included. In fact, the majority of the studies included in our analysis shows overlapping activations particularly in the STS, STS/TPJ and the MTG (see Table 5).

**TABLE 5:** Brain regions showing common activations in early blind and sighted control participants as reported by each study included in the meta‐analysis

N	First author/year	Common activations between SC and EB
1	Bedny et al. (2009)	TPJ, PFC and mPFC
2	Bedny et al. (2010)	No information provided
3	Bedny and Saxe (2012)	MTG
4	Bedny et al. (2015)	MTG
5	Dormal et al. (2018)	STS
6	Fairhall et al. (2017)	MTG, STS, FFA
7	Gougoux et al. (2009)	STS
8	Holig et al. (2014)	No information provided
9	Kitada et al. (2013)	MTG
10	Kitada et al. (2014)	MTG, EBA and supramarginal gyrus
11	Lewis et al. (2012)	pSTS/MTG
12	Ma and Han (2011)	dmPFC and the posterior cingulate cortex
13	Noppeney et al. (2003)	MTG
14	Ricciardi et al. (2009)	STG/MTG, premotor cortex, inferior and middle frontal gyri (IFG and MFG) and superior and inferior parietal lobe (SPL, IPL)
15	Striem‐Amit and Amedi (2014)	Multisensory parietal and frontal areas
16	van den Hurk et al. (2017)	Ventral‐temporal cortex (VTC)
17	Hwang and Matsumoto (2016)	mPFC, PCC, SFG/MFG, STS/TPJ and ATL

*Note*: The majority of the studies reported a common activation in the superior temporal sulcus (STS), temporo‐parietal junction (TPJ) and middle temporal gyrus (MTG).

In order to shed light on a possible common neural pattern of activation in sighted and blind individuals during social tasks, we performed a third meta‐analysis with both sighted and early blind individuals (33 experiments included, with 607 foci in 479 subjects). This additional analysis revealed consistent activation in the right pSTS, alongside the TPJ, and MTG during the representation of others, regardless of the group (sighted vs. early blind) (see Table 6 and Figure 2d).

**TABLE 6:** Neural bases of others' representation, regardless of visual experience

Cluster #	Cluster size (mm^3^)	Brain region	*x*	*y*	*z*
1	6,048	Right superior temporal sulcus/temporo‐parietal junction	48	−56	20
		Right superior temporal gyrus	64	−22	6
		Right superior temporal gyrus	58	−12	2
		Right middle temporal gyrus	62	−38	8
		Right superior temporal gyrus	56	−32	10
		Right superior temporal gyrus	66	−22	−4
		Right inferior temporal gyrus	56	−62	−2
		Right inferior temporal gyrus	54	−68	2
		Right superior temporal sulcus/temporo‐parietal junction	58	−50	6

*Note*: From left to right, the table reports the size (in mm^3^), stereotaxic coordinates of local maxima and anatomical labeling of the clusters which were consistently associated with representing others regardless of the group.

### 3.4 Others' representation in early blind versus sighted control individuals

In the early blind groups the processing of others was associated with stronger consistent bilateral activity in left fusiform gyrus and in the left middle temporal cortex compared to sighted individuals (see Table 4 and Figure 2c). The reverse comparison highlighted the right middle/superior temporal gyrus (see Table 4 and Figure 2c).

## 4 DISCUSSION

The study of the neural bases of social cognition in the blind brain has been somehow neglected, with only a few studies specifically investigating whether and how the lack of visual input affects the functional architecture of the “social brain.” Some studies showed similar patterns of brain activity in early blind and sighted individuals during tasks tapping on social cognition abilities (Bedny et al., 2009; Ricciardi et al., 2009), while other studies suggested that social brain networks develop differently following early visual deprivation (Gougoux et al., 2009; Holig et al., 2014). These inconsistencies reported in the neuroimaging literature on social processing in blind individuals may also reflect possible confounds associated with individual studies, for example, the influence of experimental and analytic procedures as well as that of the small sample sizes (Carp, 2012). Moreover, the effects reported by individual studies are harder to generalize to the entire target group (here, the early blind), regardless the specific procedures used (Muller et al., 2018).

In light of this, we pursued a meta‐analytic approach to isolate the most consistent results in the available literature, controlling for possible confounding effects via stringent criteria for study selection. In particular, we aimed to investigate: (a) the neural coding of others' representation in early blind individuals, and (b) the specific brain regions recruited in early blind compared with sighted control individuals, during social processing of others. Although we could count only on a low number of contrasts, preventing more detailed analyses such as a direct comparison of nodes of the social brain possibly differently engaged by auditory and haptic inputs, our sample is in line with current recommendations for ALE meta‐analyses (Eickhoff et al., 2016; Muller et al., 2018).

Our findings demonstrate that the regions typically associated with the key nodes of the AON mediate social cognition abilities in early blind individuals on the basis of non‐visual inputs, as they do during actual *observation* of others in sighted individuals (Gardner et al., 2015). In particular, we found consistent overlapping activations in the middle temporal gyrus bilaterally, alongside the left fusiform gyrus and the right superior temporal gyrus and finally in the right inferior frontal gyrus of early blind individuals during others' processing. The bilateral activation of the middle temporal gyrus has been previously reported for the AON in a quantitative meta‐analysis on more than 100 studies in sighted individuals (Caspers et al., 2010). Moreover, similar results, including right frontal activation, are reported in a meta‐analysis investigating brain regions showing mirror properties through visual and auditory modalities in sighted individuals (Molenberghs, Cunnington, & Mattingley, 2012).

Of note, we found stronger consistent responses in the AON in the early blind as compared to sighted subjects. This is not surprising, since the studies we included in the meta‐analysis mostly employed auditory inputs (~90% of the studies, only two studies using haptic stimuli) that are the typical stimuli on which blind individuals rely on in social interactions, whereas sighted individuals are mainly guided by visual cues. This may also account for the different pattern of lateralization observed in the activation of the AON in blind and sighted individuals, with the former showing more consistent activations in the left hemisphere particularly in the middle temporal gyrus and fusiform gyrus, while the reverse comparison highlighted the activation of the right part of the middle and superior temporal cortex. The different pattern of hemispheric lateralization may depend on the high familiarity that blind individuals have in recognizing human actions or emotions on the basis of auditory (and haptic) stimuli, with prior evidence suggesting that action familiarity is associated with increasing activity in left part of the AON (Gardner et al., 2015; Ricciardi et al., 2009).

An additional analysis carried out on the whole sample of participants (regardless visual experience) confirmed the common activation in a right temporal cluster, comprising the STG, the TPJ and the MTG regions. This cluster appears to be consistently engaged in both groups during social processing and activations in these regions are likely to play a more general function in the perception of socially relevant stimuli, which is not bound to visual experience (Fairhall et al., 2017). These results fit with the involvement of the right STG and TPJ in a variety of social‐cognitive processes (Bahnemann, Dziobek, Prehn, Wolf, & Heekeren, 2010; Yang, Rosenblau, Keifer, & Pelphrey, 2015), such as biological motion perception (Beauchamp, Lee, Haxby, & Martin, 2003; Grossman et al., 2000; Peelen, Wiggett, & Downing, 2006), mentalizing (Schneider, Slaughter, Becker, & Dux, 2014; Wolf, Dziobek, & Heekeren, 2010), and emotion attribution to others on the basis of both visual and auditory/verbal information (e.g., Alba‐Ferrara, Ellison, & Mitchell, 2012; Ferrari, Schiavi, & Cattaneo, 2018; Gamond & Cattaneo, 2016; Lettieri et al., 2019; Redcay, Velnoskey, & Rowe, 2016; Sliwinska & Pitcher, 2018). These data suggest a two‐stage process in which the STS underpins an initial parsing of a stream of information, whether auditory or visual, into meaningful discrete elements, whose communicative meaning for decoding others' behavior and intentions involves more in‐depth analysis associated with increased activation in the TPJ node (Arioli & Canessa, 2019; Bahnemann et al., 2010; Redcay, 2008). Ethofer et al. (2006) showed that the pSTS is the input of the prosody processing system and represents the input to higher‐level social cognitive computations, associated with activity in the action observation system (Gardner et al., 2015), as well as in the mentalizing system (Schurz, Radua, Aichhorn, Richlan, & Perner, 2014). Accordingly, using visual stimuli Arioli et al. (2018) pointed to the pSTS as the input for the social interaction network, which includes key nodes of both action observation and theory of mind networks. Thus, the STS/TPJ regions may represent a domain‐specific hub associated with the analysis of the meaning of others' actions, regardless of the stimulation modality, and highly interconnected with the action observation and the mentalizing networks.

The lack of activation of the parietal cortex in the early blind, with the parietal cortex being another key node of the AON in sighted individuals, is possibly due to the low number of studies specifically focused on hand representation in our database (see Pellijeff, Bonilha, Morgan, McKenzie, & Jackson, 2006; 3/17, see Table 1). Indeed, Caspers et al. (2010) reported that only observation of hand actions was consistently associated with activations within parietal cortex, the observation of non‐hand actions was not. Moreover, although Ricciardi et al. (2009) reported a parietal activation in blind participants during auditory presentation of hand‐executed actions, this activation was much less extensive (particularly in the superior parietal cortex) in the blind compared to sighted controls. Another possible explanation for the lack of parietal activation in the blind in our meta‐analysis (also possibly accounting for the results by Ricciardi et al., 2009) is that the activation within the parietal part of the AON may be mainly driven by object‐related representations (Caspers et al., 2010), which are not present in several of the experiments included in the present analysis. In turn, our studies focused mainly on voice processing (6/17), and in part on face/body representation (4/17), this probably being responsible for the consistent activation we reported in the fusiform gyrus. Indeed, voice processing has been found to activate the fusiform face area in blind individuals (i.e., Fairhall et al., 2017).

The findings of this meta‐analysis suggest that the AON can develop despite the lack of any visual experience, with information acquired in other sensory modalities allowing an efficient representation of other individuals as agents with specific beliefs and intentions (vs. objects moved by physical forces). These results may explain why early blind individuals are able to efficiently interact in the social context and to learn by imitation of others' (e.g., Gamond et al., 2017; Oleszkiewicz et al., 2017; Ricciardi et al., 2009). Our findings suggest that regions of the social brain may work on the basis of different sensory inputs, depending on which sensory modality is available. Moreover, our findings are consistent with the results of a recent meta‐analysis by Zhang et al. (2019) and with prior fMRI studies with blind individuals in the social domain (e.g., Bedny et al., 2009; Ricciardi et al., 2009) suggesting that brain regions that are consistently recruited for different functions in sighted individuals, such as the dorsal fronto‐parietal network for spatial function and ventral occipito‐temporal network for object function, and—as shown here—the AON for social function, maintain their specialization despite the lack of a normal visual experience. This observation on the “social blind brain” is in line with the current, more general perspective on the blind brain that undergoes a functional reorganization due to the lack of visual experience, but whose large‐scale architecture appears to be significantly preserved (e.g., Ricciardi, Papale, Cecchetti, & Pietrini, 2020).

Interestingly, we did not find evidence for any cross‐modal consistent recruitment of the occipital cortex by social tasks in this meta‐analysis. Cross‐modal plasticity typically refers to activation of the occipital cortex of the early blind in response to input acquired in other sensory modalities, like hearing and touch (for reviews, Merabet & Pascual‐Leone, 2010; Singh, Phillips, Merabet, & Sinha, 2018; Voss, 2019), and may account (at least in part) for the superior perceptual abilities of blind subjects in the spared sensory modalities (e.g., Battal, Occelli, Bertonati, Falagiarda, & Collignon, 2020; Bauer et al., 2015). Alternatively, recruitment of the occipital cortex in the blind has been proposed to also subserve high‐level (cognitive) processing (e.g., Amedi, Raz, Pianka, Malach, & Zohary, 2003; Bedny, Pascual‐Leone, Dodell‐Feder, Fedorenko, & Saxe, 2011; Lane, Kanjlia, Omaki, & Bedny, 2015) suggesting that cortical circuits that are thought to have evolved for visual perception may come to participate in abstract and symbolic higher‐cognitive functions (see Bedny, 2017). Indeed, recent evidence has shown that during high‐level cognitive tasks (i.e., memory, language and executive control tasks), there is an increased connectivity between occipital cortex and associative cortex in the lateral prefrontal, superior parietal, and mid‐temporal areas (Abboud & Cohen, 2019), with these regions being also possibly involved in social perception (Caspers et al., 2010). In line with this, we would have expected social tasks to drive activations in the occipital cortex. This was not the case. The only region that showed a sort of cross‐modal response was the fusiform face area, in the ventral stream, probably guided by a high number of studies included in our meta‐analysis focusing on voice processing (cf. Holig et al., 2014; von Kriegstein, Kleinschmidt, Sterzer, & Giraud, 2005). In this regard, it is also worth noting that haptic perception by blind individuals of facial expressions and hand shapes (Kitada et al., 2013, 2014) as well as whole‐body shape recognition via a visual‐to‐auditory sensory substitution device (SSD; Striem‐Amit & Amedi, 2014) led to activations in face and body‐dedicated circuits in the fusiform gyrus, showing that these dedicated circuits develop even in the absence of a normal visual experience. Our meta‐analysis shows that processes related to representation of others do not recruit the occipital cortex in the early blind, suggesting that differently to other cognitive tasks, social tasks may be mediated by higher‐level regions without the need to recruit additional occipital resources. Even if this might be related to the experimental heterogeneities that we highlighted above, the lack of a consistent recruitment of occipital cortex for social tasks we reported contributes to a better understanding of the functional role of “visual” areas in the blind brain.

In conclusion, our findings support the view that the brain of early blind individuals is functionally organized in the same way of the brain of sighted individuals although relying on different types of input (auditory and haptic) (see Bedny et al., 2009; Ricciardi et al., 2009). In the social domain, this may have important implications for educational programs for blind children. Early blindness may indeed predispose to features of social isolation, including autism (e.g., Hobson et al., 1999; Jure, Pogonza, & Rapin, 2016). It is therefore important to develop training administration guidelines specifically for persons with visual impairment (Hill‐Briggs, Dial, Morere, & Joyce, 2007). In this regard, an interesting approach would be to develop ad hoc auditory/haptic virtual reality social cognition trainings for children with blindness or severe visual impairment, as already employed with autistic children and young adults (e.g., Didehbani, Allen, Kandalaft, Krawczyk, & Chapman, 2016; Kandalaft, Didehbani, Krawczyk, Allen, & Chapman, 2013). Consistent evidence suggests that audio‐based virtual environments may be effective for the transfer of navigation skills in the blind (Connors, Chrastil, Sanchez, & Merabet, 2014; Sanchez & Lumbreras, 1999), and haptic virtual perception may be a valid and effective assistive technology for the education of blind children in domains like math learning (e.g., Espinosa‐Castaneda & Medellin‐Castillo, 2020). This approach—especially audio‐based virtual environments—may thus be extended to the social domain to allow the safe and non‐threatening practice of particular social skills in an educational setting. In this respect, and considering the importance for visually impaired children to study in a mainstream school (e.g., Davis & Hopwood, 2007; Parvin, 2015), school‐based social cognitive interventions on the social participation of children with blindness or severe visual impairment would be particularly critical, with teachers and peers being involved responding and reinforcing blind children' initiated interactions. A detailed description of the neuro‐cognitive processes underlying social cognition skills in blind individuals is thus critical to tailor training protocols aiming at targeting specific neuro‐cognitive functions. This may have also a translational clinical impact on the development of non‐invasive advanced SSDs able to translate social cues that are only visually available (such as face expressions, gestures and body language) to auditory or tactile feedback that can be processed by the intact social brain of visually deprived individuals, in terms of more abstract conceptual signals (see Cecchetti, Kupers, Ptito, Pietrini, & Ricciardi, 2016; Striem‐Amit & Amedi, 2014). These devices may help blind individuals in their interactions both in the physical and virtual (i.e., meetings via Skype, Meet or others) social world.

## CONFLICT OF INTEREST

The authors declare no conflict of interest to declare.

## Data Availability

Data of this study are available from the corresponding author upon request.
